# A bivariate prediction approach for adapting the health care system response to the spread of COVID-19

**DOI:** 10.1371/journal.pone.0240150

**Published:** 2020-10-15

**Authors:** Paolo Berta, Paolo Paruolo, Stefano Verzillo, Pietro Giorgio Lovaglio

**Affiliations:** 1 Department of Statistics and Quantitative Methods, University of Milan-Bicocca, Milan, Italy; 2 European Commission Joint Research Centre, Ispra, Italy; Witten/Herdecke University, GERMANY

## Abstract

The spread of COVID-19 implied a large and fast increase of demand for intensive care services. To face this increase in demand, health care systems need to adapt their response by increasing hospital beds, intensive care unit (ICU) capacity and by (re-)deploying doctors and other personnel. This paper proposes a forecast approach based on the Vector Error Correction model for the daily counts of hospitalized patients with symptoms and of patients in ICU, using publicly available data on the current COVID-19 outbreak in Italy, Switzerland and Spain. The level of analysis is the local government managing the health care system response, which corresponds to regions for Italy. The one-week-ahead forecasts are validated with out-of-sample data over successive weeks; they are found to provide timely and robust prediction of ICU capacity needs in Lombardy, the most-affected Italian region, starting from the sample of the first 2 weeks of data. The same methodology is successfully validated on other Italian regions, Switzerland and Spain. This approach may be used in other countries/regions/provinces to help adapt the health care system response to COVID-19 (or other similar disease); for this purpose, the open-source software code to produce the forecasts is provided with the paper.

## 1. Introduction

The human-to-human transmission of SARS-CoV-2 has spread around the world, prompting the World Health Organization to characterise it as a pandemic on March 11, 2020, see [1]. The European Centre for Disease Prevention and Control (ECDC) reported 2, 520, 522 infected between 31 December 2019 and April 22, 2020, including 176, 786 deaths, affecting more than 210 countries from all five continents, see the ECDC Epidemiological Update.

Several regions and countries have introduced strong measures to mitigate contagion through rigid social-distance measures and partial or total lock-downs. Italy has been the country with an early onset of the pandemic, with the region of Lombardy particularly affected by COVID-19.

Each local area experiences specificities in the spread of this epidemic: the spread of the virus is likely to differ between regions with different population density, age structure, and culture or habits influencing social proximity; similarly, different characteristics of the health care system and different approaches in testing for SARS-CoV-2 and related confinement approaches are expected to affect the diffusion of the disease. These specificities need to be accounted for in forecasting models.

Different indicators on the spread of the epidemic may have different levels of reliability. Indicators frequently reported are number of tested, infected, hospitalised, deceased and patients in intensive care units. Many of these indicators may reflect regional differences in reporting and suffer from selection bias. For instance the number of infected may be biased because of undetected or untested cases; the number of deaths due to Covid-19 may be biased by different practice in testing policies of deceased patients; see [2].

From the perspective of the health-system response to the local epidemic, the most important indicators are the number of patients requiring ICU admission, and their population at risk, associated to the hospitalized patients with COVID-19 symptoms. Both these indicators appear to be less prone to misreporting or selection bias than the number of infected, because hospitalisations are officially recorded following standardised protocols; this makes the corresponding time series more reliable, see [3]. These time series provide key information to adapt hospital facilities and staff in response to the evolution of the outbreak; see [4] for a classification of various patients groups and flows among them.

Moreover, the ECDC [5] indicated bed occupancy in ICU as the reference indicator of seriousness and of impact of the COVID-19 disease, both at community (region) and at hospital level. All these element indicate that ICU bed occupancy and its forecasts should be a central piece of information to craft the local/regional health care response, shifting or enhancing ICU capacity accordingly, [6]. This indicates a clear need for mathematical, econometric and statistical models to obtain timely short-term forecasts of ICU capacity, for local governments to plan—and quickly adjust—their health care resources.

Within the econometrics and statistics literature on COVID-19, forecasting has so far mainly focused on the evolution of infected people, deaths and hospitalized patients, including the ones in ICU. Most forecasting approaches in this area have been so far univariate, i.e. they have used the dynamics of the single time series of the outcome of interest to produce multi-step-ahead predictions.

Using only information contained in a single time series as in univariate approaches imposes limitations to the associated forecasts. One of them is caused by ignoring possible co-movements with other available time-series. Adding other time series as predictors, similarly to ARIMAX models, increases the explanatory power of the model. However, in order to use these models for multi-step-ahead predictions, one needs to forecast also the predictors; this is equivalent to a multivariate time-series approach, which is the one proposed in this paper.

For example, Remuzzi and Remuzzi [7] forecast the number of infected patients and hospitalized ICU admissions using an exponential trend model at the onset of the epidemic. Other examples use phenomenological models, [see 8, 9], exponential smoothing, trend and seasonal models [10], ARIMA models [11, 12] or state space models for disease transmission over time within Susceptible-Infectious-Recovered (SIR)-like models [13].

Unlike previous approaches, the present paper proposes a bivariate structural model to forecast the daily need of ICUs beds (*IC*) jointly with the number of patients hospitalized with COVID-19 symptoms (*HwS*), which approximates the population at risk for *IC*. In this approach, the joint dynamics of *IC* and *HwS* is modeled and used to produce multi-step-ahead predictions.

The model exploits the fact that these two time series are linked, theoretically as well as empirically; in technical language, they are cointegrated, as detailed below. The proposed forecast tool is obtained as the prediction of an estimated multivariate Vector Error Correction Model (VECM) to describe the joint dynamics of *IC* and *HwS*, see Engle and Granger [14], Johansen [15, 16].

There are three main advantages of using a VECM approach to forecast *IC*. First, it embodies the structural relation existing between the level of *IC* and the level of *HwS*: hospitalised patients may worsen their health status within a few days in hospital, and require ICU acute treatment. The *HwS* series *de facto* proxies the population at risk of treatment in ICU, and it is a natural predictor of *IC* flows.

Second, the use of *HwS* as a predictor summarises many external factors affecting ICU. In fact *IC* depends on hospital admission policies (ICU turnover, co-presence in ICU of COVID-19 and non-COVID-19 patients) and/or public health measures put in place during the epidemic (social distancing, lockdown). These factors all affect *HwS* prior to affecting *IC*, and using the information in *HwS* can help summarise the influence of all these factors on the forecast of *IC*.

Third, the VECM model presents well interpretable parameters, and it allows flexibility in the specification of the deterministic trend component. This permits to define various specifications, which can be fitted and compared. In particular, the strength of the cointegration relation, the speed of adjustment between series towards the equilibrium, the number of significant lags of the *IC* equations are all useful indications on the goodness of fit of the model. Out-of-sample forecasts can also be used to compare alternative models.

The aim of the present paper is to propose the VECM class of models as a predictive tool for the forecast of *IC* demand. Given the large class of alternative models, the present paper does not attempt to run an exhaustive forecast comparison with all alternative predictive models; this comparison is bound not to be exhaustive, both in terms of alternative models and of datasets. Rather, in line with the forecasting literature [17], it uses the Random Walk model as a benchmark reference forecast, on a selection of datasets from Italy, Spain and Switzerland.

In principle, the Random Walk benchmark allows to compare any given model with all present and future competing models and forecast techniques. This benchmark is found to be effective in the applications in the paper, as it is fund to outperform the present approach close to the flat peak of ICU demand. Note that prediction of the flat period is not troublesome, both from a health care system response viewpoint and from the methodological perspective. In fact, on the one hand, the health care system does not require immediate re-adjustments of resources. On the other hand, the proposed methodology is not expected to outperform the Random walk model when ICU demand is flat.

The bivariate nature of the proposed approach, while an advancement with respect to existing univariate time series techniques, falls short of incorporating (and predicting) the time series of other relevant population groups, such as the susceptible, infected, diagnosed, recognised, threatened, healed, ailing and extinct, see Giordano et al. [18]. This is done in micro-simulation models widely adopted by epidemiologists.

The purpose of such models is to simulate and explore the impact of non-pharmaceutical interventions to reduce (COVID-19) mortality and healthcare demand. These approaches are broadly used to predict aggregate trends in disease incidence and mortality under alternative scenarios to guide critical policy decisions. However, they typically require detailed parameterizations of high-resolution information on population density, progression of the disease and travel patterns. Examples are (area’s) average age, household distribution size, class sizes, staff-student ratios (to model transmission events) or age-stratified proportion of infections, delay from onset of symptoms hospitalisation, duration of hospitalisation, infection fatality ratio (to model the disease progression and healthcare demand), see Ferguson et al. [19]

While these detailed data are not available to the authors in the present case hence preventing the application of this approach, the multivariate nature of the proposed VECM class of models may allow extensions of the present approach to other time series representing these groups. These extensions appear a promising area for future research.

The VECM class of models is applied in this paper to the daily time series of *IC* and *HwS*, with the aim to predict *IC* one week ahead. These model could be extended to include information from lower-frequency time series, as in [20] and [21], if this type of information is available. This is however, not the case in the applications presented in this paper. Another possible alternative to the VECM class of models could be to combine forecast obtained from several econometric time series models [22, 23], also in the context of mixed frequency data [24, 25]. This option, however, would prevent interpretability of model coefficients, which appears an essential issue, especially in a health policy context. Moreover, adapting the response of the health-system requires ease-of-use and simplicity of the forecasting tool for the health authorities; both these aspect are better addressed by the present recourse to a single class of models.

This paper applies the forecast tools to the daily counts of hospitalized patients with symptoms and of patients in ICU, using open data from the current COVID-19 outbreak in Italian regions, as well data from Switzerland and Spain. The level of analysis is taken to be regions in Italy, because they are responsible for the management the health care system. Results are presented for the most affected regions in Northern Italy, such as Lombardy, Veneto, Emilia-Romagna. The proposed approach is validated also on data for Switzerland and Spain.

The out-of-sample daily forecast accuracy is presented and compared across different models within the VECM class, using standard forecasting accuracy indices, [17]. The benchmark model is taken to be the Random Walk, as often done in the literature. Results are presented both for the overall sample of data, and for a set of rolling sequence of weeks. The results show a good forecast performance for the proposed models.

## 2. Data

The outbreak in northern Italian regions had similar evolutions, which are well represented by the one of Lombardy (10 million inhabitants), which is the first and the most affected Italian region. Many hospitals in Lombardy have undergone dramatic changes after the onset of COVID-19: many wards have been reallocated to host COVID-19 patients, outpatients activities suspended, elective surgeries postponed, new hirings of doctors and nurses.

The first Italian patient tested positive on February 20, 2020 in Lombardy [see 4]. The COVID-19 ICU Lombardy Network was established on February 21 to coordinate the critical care response to the outbreak, with the identified main priority to increase ICU capacity. The Italian Civil Protection started publishing daily data at national, regional and provincial level on the evolution of the COVID-19 contagion in the country from February 24, 2020, see the dedicated Github repository, including numbers of infected, patients hospitalized with COVID-19 symptoms and patients in ICU. The initial ICU capacity of 842 beds in Lombardy was increased to approximately 1755 by the middle of April.

The Italian national government and the one of Lombardy adopted successive restrictive measures to contain the spread of the virus, with increasing intensity of social distancing. The major decrees are dated March 1 (“Decreto Zone Rosse”—red-zones Decree), March 9 (“Decreto #IoRestoaCasa”—#IStayHome Decree) and March 22 (“Decreto Chiusa Italia”—Locked-Italy Decree).

**Fig 1:**
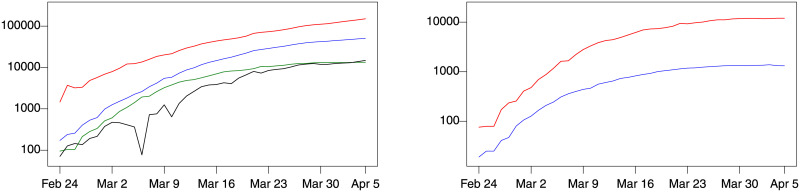
Lombardy data, February 24, 2020 to April 5, 2020. Left: Number of hospitalized patients (green), home-quarantined (black), tested (red), infected (blue). Right: Patients hospitalized with symptoms (red, *HwS*) and admitted in ICU (blue, *IC*). Both vertical axes are logarithmic, with successive ticks corresponding to increasing powers of 10.

Fig 1 shows the time evolution of the main indicators for Lombardy. The number of tested and of infected patients grows exponentially, the number of patients needing hospitalization grows rapidly, causing distress to the health care system. The right panel of Fig 1 shows the increase of patients hospitalized with symptoms (*HwS*) and of inpatients in ICU (*IC*).

The right panel in Fig 1 plots the time series of *ic* and *hws* (the log of *IC* and *HwS*). Note the parallel trend between the two, which suggests that the two may have a common trend. This is the same as saying that they are contegrated in the sense of Engle and Granger [14], i.e. that there exists a linear combination of the two that has no trend. The prediction of *IC* for Lombardy is the main empirical application of the present paper.

The time series of other Italian regions corresponding to Fig 1 show similar patterns, albeit with a different starting date; they are not graphed here for brevity. Similar patterns are also observed for data from Spain and Switzerland. The Italian regions of Veneto and Emilia-Romagna, as Spain and Switzerland, are used to validate the proposed approach.

The available data for Lombardy covers the period February 24–April 5 2020, split into a training sample (February 28–March 29) and an out-of-sample period (March 30–April 5). Data from February 24 to 27 was used to initialize differences and lags, i.e. as pre-estimation period. This sample and its divisions are called the ‘full sample’ in the following. A lag length of *k* = 4 was used, resulting in 3 lagged changes appearing in Eq (1). The pre-estimation period was chosen so as to align the estimation sample in the first and second stage.

## 3. Method

The forecasts are produced using the VECM. The VECM is a special case of the Vector AutoRegressive (VAR) models, and allows for the presence of stationary and nonstationary variables, see Hendry [26]. VECMs incorporate one (or more) underlying long-run equilibrium relation(s) among the time series, called the cointegration relation(s), as well as adjustment dynamics that corrects towards equilibrium. Finally, they allow the growth rates of lagged variables to influence each other.

### 3.1 Vector error correction models

The nature of the trends in the system can be both deterministic, i.e. a function of time, and stochastic, as represented by random walks. A *p* × 1 vector *x*_*t*_ of variables observed at times *t* = 1, 2, … that is stationary in its first differences Δ*x*_*t*_ = *x*_*t*_ − *x*_*t*−1_ is called integrated of order 1, or I(1). The vector *x*_*t*_ is generated by a VECM if it satisfies Eq (1) below

$$\begin{array}{c} \Delta x_{t}=\alpha \beta^{\prime }x_{t-1}+\sum_{j=1}^{k-1}\Gamma_{j}\Delta x_{t-j}+\mu D_{t}+\varepsilon_{t}, \end{array}$$ (1)

where *ε*_*t*_ is a *p* × 1 vector of random variables (not necessary Gaussian), i.i.d. over time, with mean 0 and positive variance-covariance matrix Ω. Here Δ*x*_*t*_ = (Δ*x*_1,*t*_, …, Δ*x*_*p*,*t*_)′ is the *p* × 1 vector of the first differences, *D*_*t*_ is an *n*_*D*_×1 vector of deterministic variables; *D*_*t*_ is taken here to include a constant 1 and a linear trend *t*. The Γ_*j*_ are *p* × *p* parameter matrices with *i*, *ℓ* element Γ_*j*,*iℓ*_ describing the influence of Δ*x*_*ℓ*,*t*−*j*_ on Δ*x*_*i*,*t*_.

The term *β*′*x*_*t*−1_ cointains *r* (in the present case *r* = 1) cointegrating relations among *x*_*t*−1_, and *α* measures adjustment towards equilibrium. Eq (1) is referred to as the VECM, in reference to the adjustment mechanism. The long-run equilibrium (cointegrating) relation *β*′*x*_*t*−1_, properly corrected for *D*_*t*_, is stationary, so that (1) provides a balanced equation: the l.h.s. is stationary, similarly to the terms *ε*_*t*_, *β*′*x*_*t*−1_ and Δ*x*_*t*−*j*_ on the r.h.s., possibly corrected for *D*_*t*_.

When *r* = 1, and the long-run equilibrium relation *β*′ in (1) is normalised setting the coefficient to *x*_1*t*−1_ equal to 1, *β*′ = (1, −*β*_2_)′, the cointegrating relation can also be written as $\beta^{\prime }x_{t-1}=x_{1t-1}-\beta_{2}^{\prime }x_{2,t-1}$. Hence, correcting for *D*_*t*_,

$$\begin{array}{c} x_{1,t-1}=\beta_{2}^{\prime }x_{2,t-1}+\varphi^{\prime }H_{1}^{\prime }D_{t}+w_{t-1}. \end{array}$$ (2)

The *β*_2_ coefficients (only one coefficient in the present case) describe the proportionality factor between *x*_1*t*_ and *x*_2*t*_ in the long-run, where in (2) *x*_1*t*_ and *x*_2*t*_ may be trending (nonstationary) while their deviation *w*_*t*−1_ is stationary. In (2), $H_{1}^{\prime }$ is a design matrix that can select a subset of *D*_*t*_ in $H_{1}^{\prime }D_{t}$.

### 3.2 Estimation

Estimation of (1) can be done in two stages following Engle and Granger [14]. The first stage is performed by estimating (2) by regression of *x*_1*t*−1_ on *x*_2,*t*−1_ and $H_{1}^{\prime }D_{t}$; this generates ${\hat{\beta }}^{\prime }x_{t-1}$ or, equivalently ${\hat{w}}_{t-1}$. These quantities are substituted in (1) in the second stage, which estimates the remaining parameters by regression of Δ*x*_*t*_ on *β*′*x*_*t*−1_, Δ*x*_*t*−*j*_ for *j* = 1, …, *k* − 1 and $H_{2}^{\prime }D_{t}$, where $H_{2}^{\prime }$ is a design matrix that selects a subset of *D*_*t*_ in $H_{2}^{\prime }D_{t}$.

The specification of the deterministic terms in $H_{m}^{\prime }D_{t}$, *m* = 1, 2, in the two stages is labeled *d*(*i*, *j*) in the following, where *i* refers to the first stage and *j* to the second one. Consider first *i*; when *i* = 2, both the constant and the trend *t* are included in $H_{1}^{\prime }D_{t}$, while when *i* = 1 only the constant is included. Finally when *i* = 0, no deterministic component is included in (2). The same convention is used for *j* in relation to $H_{2}^{\prime }D_{t}$ and (1). These models are the ones proposed in Johansen [16] (eq. (5.13) to (5.17), page 81); more details on the properties of the model are provided in Berta et al. [27].

The time series of *ic* and *hws* are clearly non-stationary and apparently show the same common trend (Fig 1). This motivates the use of the VECM class of models *d*(*i*, *j*) introduced above. Two of these models appeared of special relevance, namely the *d*(2, 1) and *d*(1, 1) models, because they both are characterized by growth rates with constant and possibly non-zero expectations, see Berta et al. [27].

When *x*_1,*t*_ = *ic*_*t*_ (the log of *IC*_*t*_) and *x*_2,*t*_ = *hws*_*t*_ (the log of *HwS*_*t*_), the interpretation of the coefficients is as follows. The cointegrating relation (2) reads *ic*_*t*−1_ = *β*_2_ *hws*_*t*−1_ + *a*_0_ + *a*_1_ *t* + *w*_*t*−1_ for *j* = 2, where for *j* = 1, *a*_1_ = 0 and for *j* = 0, *a*_1_ = *a*_2_ = 0. Taking exponentials, $IC_{t-1}=\gamma_{t}HwS_{t-1}^{\beta_{2}}\;\eta_{t-1}$, where *γ*_*t*_ = exp(*a*_0_ + *a*_1_*t*) is the coefficient of proportionality and *η*_*t*_ = exp(*w*_*t*_) is an error term.

This relation is a relation of semi-proportionality when 0 < *β*_2_ < 1 i.e. *IC* moves less than proportionally with respect to *HwS*. On the contrary, when *β*_2_ = 1, *IC* are directly proportional to *HwS*. The constant of proportionality *γ*_*t*_ equals 1 when *a*_0_ = *a*_1_ = 0. When *a*_1_ ≠ 0, *γ*_*t*_ changes over time; this reflects shifts in the transition to ICU admission from symptomatic status, as well as possibly the severity of the disease.

The adjustment Eq (1) describes how changes of *x*_1,*t*_ = *ic*_*t*_ and *x*_2,*t*_ = *hws*_*t*_ adjust to deviations *w*_*t*_ (equilibrium-correction), as well as to past changes. The analysis of the *i*, *ℓ* coefficients in Γ_*j*,*iℓ*_ describing the influence of Δ*x*_*ℓ*,*t*−*j*_ on Δ*x*_*i*,*t*_ allows to verify if the dynamics of *x*_2,*t*_ is autonomous, and how it affects Δ*x*_1,*t*_ via its lagged values Δ*x*_2,*t*−*j*_.

### 3.3 Forecast performance

The forecast performance of the model is evaluated by means of standard forecast accuracy indices, namely the Mean Absolute Error (MAE), ${\text{MAE}}_{i}=h^{-1}\sum_{j=1}^{h}\left|y_{t+j}-y_{t+j|t}^{\left(i\right)}\right|$, and Mean Absolute Percentage Error (MAPE), ${\text{MAPE}}_{i}=h^{-1}\sum_{j=1}^{h}|y_{t+j}-y_{t+j|t}^{\left(i\right)}\left|/\right|y_{t+j}|$, where predictions are made at time *t*, *y*_*t* + *j*_ indicates the time series to be predicted at time *t* + *j*, and $y_{t+j|t}^{\left(i\right)}$ its *i*-th predictor based on information up to time *t*; see e.g. [17].

Because the forecast are made on the log of the *IC*_*t*_ series, its MAE also corresponds to ‘ln Q’ metric indicator of Tofallis [28] and provides an approximation to the MAPE for the corresponding *IC*_*t*_ forecast. In fact, one has |log(*a*_*t*_/*b*_*t*_)| ≈ |(*a*_*t*_ − *b*_*t*_)/*b*_*t*_| where the approximation hold for |(*a*_*t*_ − *b*_*t*_)/*b*_*t*_| (possibly much) lower than 1, where *a*_*t*_ is the predictor of *IC*_*t*_ and *b*_*t*_ is *IC*_*t*_. This implies that the average of |log(*a*_*t*_/*b*_*t*_)| (i.e. the MAE for forecasts of *ic*_*t*_ = log *IC*_*t*_) is approximately equal to the average of |(*a*_*t*_ − *b*_*t*_)/*b*_*t*_|, (which is the MAPE for forecasts of *IC*_*t*_), when the predictor of *IC*_*t*_ is constructed from the one of *ic*_*t*_ = log *IC*_*t*_.

Moreover, to allow comparability with other predictions, the forecast accuracy indices of the proposed models are compared with the ones for the Random Walk model, which is often used as the benchmark in the forecast literature, see e.g. [17]. Specifically, the relative version of MAE and MAPE are labelled MAER and MAPER in the following; they equal MAER_*i*_ = MAE_*i*_/MAE_0_ and MAPER_*i*_ = MAPE_*i*_/MAPE_0_, where the index 0 corresponds to the Random Walk. Values of MAER_*i*_ and MAPER_*i*_ below one are indication of better performance of model *i* as compared with the Random Walk.

A practical procedure to select a forecast model is to estimate all models on the available (rolling or full) estimation sample(s), retaining one week of data for out-of-sample forecast comparison. If a preferred model is needed, one can choose the model that gives minimal MAE and MAPE in the out-of-sample forecasts for the last week.

To better validate estimates and to assess the flexibility of the proposed models, a set of rolling training samples and out-of-sample periods were considered. The first training sample covered the first two weeks of data, i.e. from February 24 to March 8 for Lombardy; out-of-sample forecast were made for the following seven days. Additional training samples of data were considered, each one obtained by adding one extra week of data. The last out-of-sample forecast exercise coincides with the full training sample. In case more than three weeks of data were available, the first week of data was discarded to allow for a ‘burn-in’ period. The same setup of full sample and rolling samples analyses was used for the other Italian regions, Switzerland and Spain.

## 4. Results

The models *d*(*i*, *j*) described in the previous section were applied to data on *x*_1,*t*_ = *ic*_*t*_ and *x*_2,*t*_ = *hws*_*t*_ from Italian regions, Switzerland and Spain. Results are presented in this section, starting with Lombardy.

### 4.1 Model estimation for Lombardy

Table 1 reports estimates of the model parameters for the *d*(1, 1), which is selected here as the *d*(*i*, *j*) model with lowest MAE and MAPE over this sample, as detailed in the following susection.

**Table 1:** Estimates for the *d*(1, 1) model for Lombardy, estimation period March 2 to March 29 2020.

Engle Granger first stage, Eq (2)										
Cointegrating relation: ${\hat{w}}_{t-1}=ic_{t-1}-0.74hws_{t-1}-0.24$										
Engle Granger second stage, Eq (1)										
	eq Δ*ic*_*t*_					eq Δ*hws*_*t*_				
predictors	coeff	se	*t*-ratio	*p*-value		coeff	se	*t*-ratio	*p*-value	
${\hat{w}}_{t-1}$	-0.28	0.11	-2.5	0.01	**	0.69	0.24	2.81	0.00	***
Δ*ic*_*t*−1_	-0.02	0.13	-0.12	0.90		-0.66	0.29	-2.33	0.02	*
Δ*hws*_*t*−1_	-0.04	0.08	-0.53	0.59		0.18	0.18	1.02	0.31	
Δ*ic*_*t*−2_	0.16	0.06	2.59	0.01	**	0.26	0.13	1.97	0.05	*
Δ*hws*_*t*−2_	0.07	0.06	1.18	0.24		0.30	0.12	2.42	0.02	*
Δ*ic*_*t*−3_	0.38	0.06	6.27	0.00	***	0.44	0.13	3.32	0.00	***
Δ*hws*_*t*−3_	0.18	0.05	3.78	0.00	***	0.24	0.11	2.22	0.03	*
1	0.00	0.01	-0.44	0.66		0.00	0.02	0.01	0.99	

*, **, ***: significant at 5%, 1%, 0.1% level.

Several remarks are in order. The *β*_2_ coefficient is estimated equal to 0.74, showing a less-than-proportional relations between *HwS* and *IC*. The constant of proportionality *γ*_*t*_ is approximately 1.27, higher than 1. The adjustment of Δ*ic*_*t*_ to the disequilibrium error is strong and significant, (−0.28). The one of Δ*hws*_*t*_ is larger in magnitude and also significant. This indicates that both Δ*ic*_*t*_ and Δ*hws*_*t*_ adjust towards equilibrium.

The analysis of the Γ_*j*,*iℓ*_ coefficients for the equation of IC patients shows that the growth rates of *IC* and *HwS* lagged 2 or 3 days significantly help to predict the growth rate of *IC*. This shows the value added of the multivariate approach over univariate ones, where *HwS* helps to predict *IC* both because it enters the equilibrium-correction term and via the growth rates lagged 2 or 3 days. Note that the dynamic equation for *HwS* is also useful in computing multi-step-ahead predictions for *IC*.

**Fig 2:**
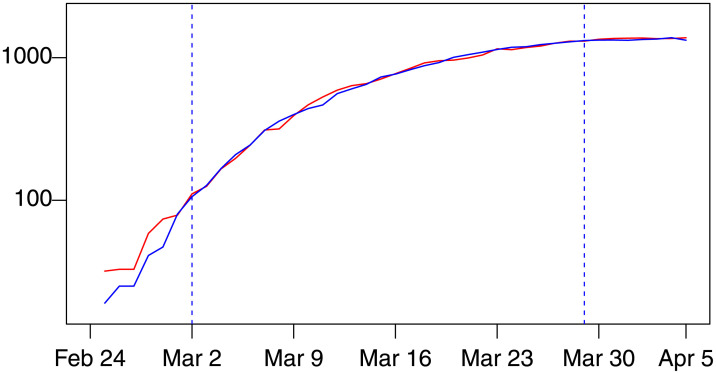
Actual *ic*_*t*_ (blue) and fitted values 0.74*hws*_*t*−1_ + 0.24 (red) from the first stage regression of the *d*(1, 1) model for Lombardy, estimation period February 28–March 29, 2020. The graph also includes the out-of-sample periods of February 24 to March 1 (pre-estimation period) and from March 30 to April 5, 2020, where these periods are separated by vertical dashed lines.

The fit of the corresponding first stage regression is plotted in Fig 2. The graph also includes the pre-estimation sample between February 24 to March 1 and the out-of-sample forecast period from March 30 to April 5, 2020. Note that Fig 2 represents a cross-section relation and not a predictive dynamic equation; in fact the contemporaneous value of *hws*_*t*_ appears as an explanatory variable. This equation represents, instead, the equilibrium relation between *ic*_*t*_ and *hws*_*t*_ towards which Δ*ic*_*t*_ adjusts via the equilibrium correction Eq (1).

**Fig 3:**
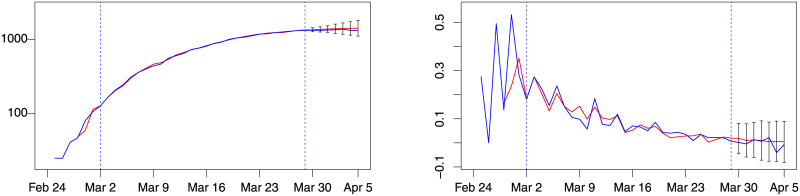
Lombardy forecast for week 6, model *d*(1, 1). Blue: actual; red: fitted (in estimation sample) or forecast (out-of-sample). Left: number of patients admitted in ICU. Right: % change of patients admitted in ICU. Estimation sample: March 2–March 29, between dashed vertical lines. Out-of-sample period: March 30–April 5. Forecast intervals ± 3 standard errors.

The *d*(1, 1) model was then used to forecast *ic*_*t*+*j*_ setting *t* at March 29, 2020, and considering *j* = 1, …, 7, which correspond to March 30–April 5, 2020. The forecasts of *ic*_*t* + *j*_ and Δ*ic*_*t* + *j*_ are graphed in Fig 3, respectively in the left and right graphs; the corresponding out-of-sample forecast intervals, obtained as ± 3 times the model forecast standard error, are also reported. The model graphically shows a good forecast performance.

### 4.2 Forecasting accuracy for Lombardy

The MAE and MAPE indices—as well as their relative versions with respect to the Random Walk MAER and MAPER— were calculated in the out-of-sample period both for models *d*(1, 0), *d*(1, 1), *d*(2, 0), *d*(2, 1); results are reported in Table 2. In the out-of-sample period of March 30 to April 5, MAE and MAPE are smallest for the *d*(1, 1) model, which is hence the preferred model. The absolute forecast percentage error for this model of *IC*_*t*_ is approximately 4%.

**Table 2:** Mean Absolute Error (MAE), Mean Absolute Percentage Error (MAPE) along with their relative versions with respect to the Random Walk model (MAER and MAPER, respectively) for models *d*(*i*, *j*) over different training samples and forecast periods.

forecast week:	week 3				week 4			
model:	*d*(1, 0)	*d*(1, 1)	*d*(2, 0)	*d*(2, 1)	*d*(1, 0)	*d*(1, 1)	*d*(2, 0)	*d*(2, 1)
MAE	**0.04**	0.64	0.34	0.56	0.04	0.09	**0.01**	0.06
MAER	**0.10**	1.62	0.87	1.42	0.15	0.35	**0.06**	0.25
MAPE	**0.01**	0.10	0.05	0.09	0.01	0.01	**0.00**	0.01
MAPER	**0.09**	1.49	0.80	1.30	0.15	0.33	**0.06**	0.24
forecast week:	week 5				week 6			
model:	*d*(1, 0)	*d*(1, 1)	*d*(2, 0)	*d*(2, 1)	*d*(1, 0)	*d*(1, 1)	*d*(2, 0)	*d*(2, 1)
MAE	**0.05**	0.06	0.08	0.08	0.05	**0.04**	0.06	0.05
MAER	**0.55**	0.62	0.79	0.86	4.16	**3.26**	5.32	4.38
MAPE	**0.01**	0.01	0.01	0.01	0.01	**0.01**	0.01	0.01
MAPER	**0.54**	0.61	0.78	0.85	4.16	**3.26**	5.32	4.38

**Bold** entries correspond to the model with smallest MAE or MAPE for the given forecast week.

The rolling out-of-sample performance of the models is also reported in Table 2, which shows that the best performance was obtained by the *d*(1, 0) model for weeks 3 and 5, model *d*(2, 0) for week 4 and model *d*(1, 1) for week 6. The level of absolute forecast percentage error of *IC*_*t*_ for the best models is between 1% and 5% across all weeks.

It can be noted that models with fewer parameters work best in the early weeks, where there is less information available. The best specification of the deterministic component appears to change over weeks; this suggest that more flexible specifications of this component may be useful over (future) longer spans of data.

**Fig 4:**
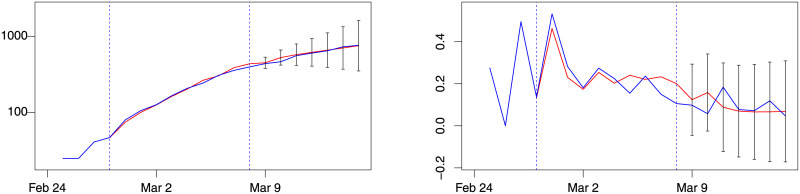
Lombardy forecast for week 3, model *d*(1, 0). Blue: actual; red: fitted (in estimation sample) or forecast (out-of-sample). Left: number of patients admitted in ICU. Right: % change of patients admitted in ICU. Training sample: February 28–March 8. Out-of-sample period: March 9–15. Forecast intervals ± 3 forecast standard errors.

**Fig 5:**
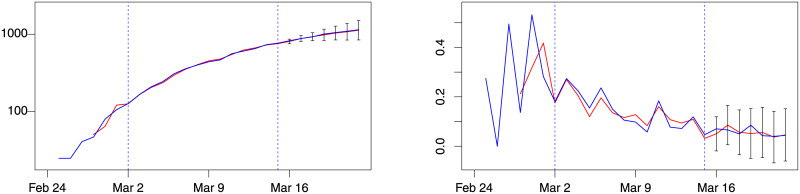
Lombardy forecast for week 4, model *d*(2, 0). Blue: actual; red: fitted (in estimation sample) or forecast (out-of-sample). Left: number of patients admitted in ICU. Right: % change of patients admitted in ICU. Training sample: February 28–March 15. Out-of-sample period: March 16–21. Forecast intervals ± 3 forecast standard errors.

**Fig 6:**
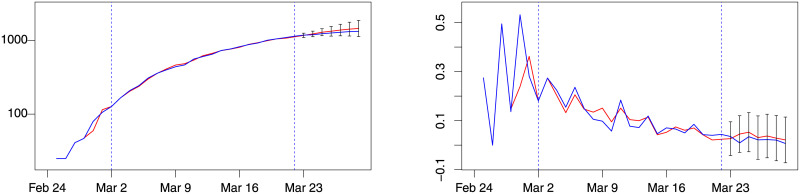
Lombardy forecast for week 5, model *d*(1, 0). Blue: actual; red: fitted (in estimation sample) or forecast (out-of-sample). Left: number of patients admitted in ICU. Right: % change of patients admitted in ICU. Training sample: February 28–March 22. Out-of-sample period: March 23–29. Forecast intervals ± 3 forecast standard errors.

Overall, both the absolute forecast error (MAE) and the absolute relative forecast error (MAPE) of the best models are small, indicating a satisfactory forecast accuracy. The out-of-sample performance of the best model for weeks 3, 4 and 5 for Lombardy are reported in Figs 4, 5 and 6.

Table 2 also reports MAER and MAPER. It can be observed that the best *d*(*i*, *j*) model outperforms the Random Walk model expect for week 6, where the level evolution of the *IC*_*t*_ time series is flat, and the flat prediction of the Random Walk has lower absolute average forecast errors. Note that the level of MAE and MAPE of the best *d*(*i*, *j*) model in weeks 6 is 4%, within the range of values observed in previous weeks (1% to 5%). This shows that week 6 is the period where the Random Walk has an improved forecast performance rather than a period of lower performance of the best *d*(*i*, *j*) model.

### 4.3 External validation

**Fig 7:**
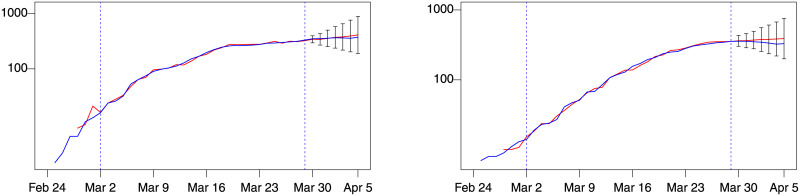
Emilia Romagna (model *d*(1, 1)) and Veneto (model *d*(2, 1)). Blue: actual; red: fitted (in estimation sample) or forecast (out-of-sample). Left: number of patients admitted in ICU in Emilia Romagna. Right: number of patients admitted in ICU in Veneto. Training sample: February 28–March 29. Out-of-sample period: March 30–April 5. Forecast intervals ± 3 standard errors.

Validation of the models was performed considering the data available at the time of writing (April 2020) for the other Italian regions of Emilia Romagna and Veneto, as well as for Spain and Switzerland. For Emilia Romagna and Veneto the forecast exercise is performed in week 6. The best *d*(*i*, *j*) models are *d*(1, 1) for Emilia Romagna and *d*(2, 1) for Veneto in term of MAE and MAPE. Fig 7 plots the level forecasts for Emilia Romagna and Veneto of these model. Both show a good forecast performance.

**Fig 8:**
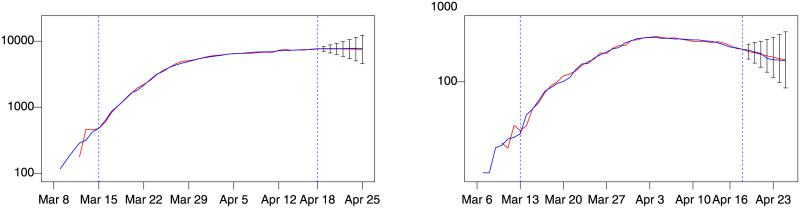
Spain (model *d*(2, 1)) and Switzerland (model *d*(1, 1)). Blue: actual; red: fitted (in estimation sample) or forecast (out-of-sample). Left: number of patients admitted in ICU in Switzerland. Right: number of patients admitted in ICU in Spain. Training sample within dashed vertical lines. Forecast intervals ± 3 standard errors.

A further validation of the model was performed considering the data for Switzerland and Spain until April 25. The best *d*(*i*, *j*) models are *d*(2, 1) for Spain and *d*(1, 1) for Switzerland in term of MAE and MAPE. Fig 8 shows forecasts of these models, again showing a good forecast performance.

## 5. Conclusions

Unlike other methodologies and approaches used for analyzing COVID-19 data, the VECM approach proposed here relies on real-time daily official data as inputs, exploits the multivariate nature of the structural relationship between *IC* and *HwS*, and can be estimated over relatively short samples. Moreover, the model adapts over time not only to the evolution of contagion, but also to a number of local regional characteristics, such as hospital access policies and containment measures, as both are typically reflected in the time series of hospitalized patients with symptoms.

In the estimated VECM for Italian regions, parameters are significant and consistent with *a priori* expectations: the *IC* series evolves over time adjusting to its past values, to deviations from the log run equilibrium with *HwS* and to the lagged values of *HwS*. The level cointegrating relation often show an overall less-than-proportional relationship between *HwS* and *IC*.

The present study has some limitations. It focuses on the available data, provided at regional level for ICU and hence ignores local (cities, provinces) variation. This prevents to link the evolutions of the analyzed time series with local conditions (local distribution of age, co-morbidities) and/or to the distribution of hospitals’ characteristics. Moreover, the models and related forecasts do not consider issues related to reaching or approaching the threshold of maximum ICU capacity; this was due both to lack of available data on the thresholds and to the dynamic adaptation of the capacity to the increased needs induced by the pandemic in the case of Italy.

Despite these limitations, the VECM forecasting tool is found to offer reliable and timely forecasts for various Italian regions including Lombardy, Veneto and Emilia-Romagna. These results are confirmed by the validation on Spanish and Swiss aggregate data.

The reliability of the forecasts was confirmed also using different small incremental samples, where forecasts seven days ahead are found to be reliable already using only two weeks of training data since the onset of contagion. Forecast accuracy largely improves with increased availability of data. It was also found that flexible specifications of the deterministic component become increasingly useful using longer spans of data.

These forecasts appear important tools for health care system managers, who need to predict the needs of ICU beds in a fast way in the fast-rising upwards phase, allowing them to calibrate and adapt the response of the health care system to the epidemic (modifying staff resources, hospital facilities and resources), in a timely fashion.

The approach proposed in this paper may be used in other countries/regions to provide local health authorities with real-time forecast numbers of ICU beds, helping to shape the health care response and to prevent shortage of hospital resources (beds, ventilators, doctors and nurses), [19].

A free software program (in the form of a public *R* package called ‘Presize’, available here, is provided with this article. This can facilitate replication the proposed analysis in other countries, regions or provinces, even at higher granularity than the regional level, if appropriate in terms of health-system decision-making and if data are available.
